# Pregabalin and Tranexamic Acid Evaluation by Two Simple and Sensitive Spectrophotometric Methods

**DOI:** 10.1155/2015/241412

**Published:** 2015-03-19

**Authors:** Nawab Sher, Nasreen Fatima, Shahnaz Perveen, Farhan Ahmed Siddiqui, Alisha Wafa Sial

**Affiliations:** ^1^Department of Chemistry, Faculty of Science, University of Karachi, Karachi 75270, Pakistan; ^2^PCSIR Laboratories Complex Karachi, Shahrah-e-Dr. Salimuzzaman Siddiqui, Karachi 75280, Pakistan; ^3^Faculty of Pharmacy, Federal Urdu University of Arts, Science and Technology, Karachi 75300, Pakistan; ^4^Faculty of Medicine, Ziauddin University, Karachi 75600, Pakistan

## Abstract

This paper demonstrates colorimetric visible spectrophotometric quantification methods for amino acid, namely, tranexamic acid and pregabalin. Both drugs contain the amino group, and when they are reacted with 2,4-dinitrophenol and 2,4,6-trinitrophenol, they give rise to yellow colored complexes showing absorption maximum at 418 nm and 425 nm, respectively, based on the Lewis acid base reaction. Detailed optimization process and stoichiometric studies were conducted along with investigation of thermodynamic features, that is, association constant and standard free energy changes. The method was linear over the concentration range of 0.02–200 *µ*gmL^−1^ with correlation coefficient of more than 0.9990 in all of the cases. Limit of detection was in range from 0.0041 to 0.0094 *µ*gmL^−1^ and limit of quantification was in the range from 0.0137 to 0.0302 *µ*gmL^−1^. Excellent recovery in Placebo spiked samples indicated that there is no interference from common excipients. The analytical methods under proposal were successfully applied to determine tranexamic acid and pregabalin in commercial products. *t-test* and *F* ratio were evaluated without noticeable difference between the proposed and reference methods.

## 1. Introduction

**Figure 1:**
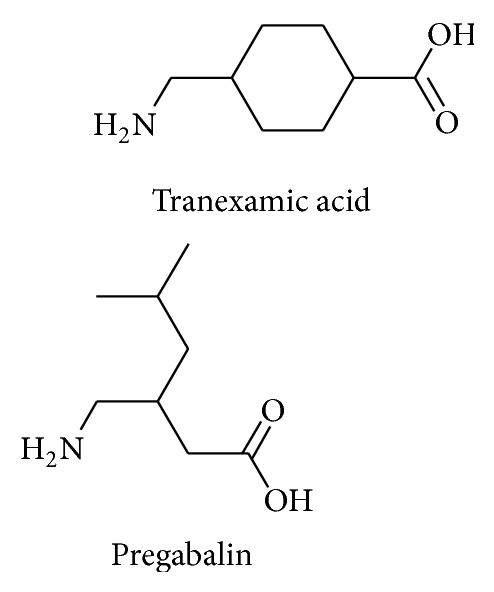
Structure of PG and TXA.

Pregabalin (PG) chemically is 3-amino methyl hexanoic acid, with the chemical formula C_8_H_17_NO_2_. Its structural and pharmacological features correspond to the mammalian neurotransmitter gamma-aminobutyric acid (GABA), and it is primarily used as anticonvulsant drug. However, its effects are much broader being analgesic, antiepileptic, antidiabetic, and anti-inflammatory drug. Further more, it has been recommended for gastrointestinal damage, alcoholism, and insomnia [1]. Exact mechanism is not known; however, it has been an established fact that it binds to calcium channel in the central nervous system which decreases calcium influx at nerve endings and therefore reduces the liberation of several associated neurotransmitters which subsequently results in the above-mentioned activities [2]. Tranexamic acid (TXA) is chemically designed as* trans*-4-(aminomethyl) cyclohexanecarboxylic acid, with chemical formula C_8_H_14_NO_2_. It is the closed cyclic analogous structure of lysine. TXA has been very potent antifibrinolytic agent, vastly used in haemorrhagic diseases. Its significant use is to treat ovarian tumors and it is recommended to manage pregnancy and reduce blood lose in surgery. It is considered as the best substitute to surgery in cases of menorrhagia [3, 4]. Chemical structure of TXA and PG is shown in Figure 1.

PG is not yet part of any pharmacopoeial monograph. However, analytical method reported for PG includes spectrophotometry [5–9], spectrofluorimetry [10], and high pressure liquid chromatography with UV detection [11], with mass detection [12], and with fluorescence detection [13]. TXA quantification has been reported through colorimetric spectrophotometry [14–18], HPLC [19], and spectrofluorometry [20]. Although chromatographic method offers high level of selectivity and specificity, the associated expenses are alarming and cannot be shouldered by every pharmaceutical industry. Moreover, the molecules of TXA and PG lack UV absorbing chromospheres, so in case of HPLC high load on column will deteriorate and reduce its life which turned out to be huge burden on low and middle economy-based industries. Amino acids like TXA and PG are highly polar and cannot be easily volatilized, so gas chromatographic method would not be easy or straightforward. Spectrofluorometers are not the common instruments in all the labs. All these suggest that the proposed analytical methods are highly important from quality control point of view. The colorimetric reagents used in this study are trinitrophenol (TNP) and dinitrophenol (DNP). These reagents have wide range utility as quality colorimetric analyzing agents in pharmaceutical industry [21–27].

A glance over the literature revealed few reports regarding charge transfer complexes for PG and TXA. A structural analogous of PG and TXA gabapentin has been determined by colorimetry-visible spectrophotometry by reacting with iodine, chloranil, chloranilic acid, 2,3-dichloro-5,6-dicyano-1,4-benzoquinone, tetracyanoethylene, methyl orange, hydroxy benzaldehyde, picric acid and with ninhydrin [28–30]. Colorimetric spectrophotometric methods for PG include reaction with 1,2-naphthoquinone-4-sulphonate sodium and 2,4-dinitrofluorobenzene [5], ascorbic acid and salicylaldehyde [6], 7,7,8,8-tetracyanoquinodimethane, 2,3-dichloro-5,6-dicyano-1,4-benzoquinone, 2,5-dichloro-3,6-dihydroxy-1,4-benzoquinone, tetracyanoethylene and 2,3,5,6-tetrachloro-1,4-benzoquinone [7], quinalizarin and alizarin [8], and vanillin, acetyl acetone, and formaldehyde [9]. Colorimetric quantification reagent for TX includes ninhydrine [14, 15], ferric chloride [15], 1,2-naphthoquinone-4-sulphonate sodium [16], ascorbic acid [17], and Azo dye [18].

There is no report on TXA and PG determination which utilizes TNP or DNP reagents. Most of the reported spectrometric methods are insensitive or need complicated extractions and heating/cooling procedures or use reagents which do not produce linear response. Moreover, most of the methods are based on the absorbance in near UV region and thus specificity is questioned. The method under proposal is highly selective and extremely simple, so it can be usefully adopted for routine analysis in quality control laboratories. The proposed methods are accompanied by the reaction of amine group of amino acid with hydroxyl group, activated by neighboring nitro groups of both TNP and DNP. As a result, yellow color complexes are formed on simple addition of the two reagents and no extra process is required. Thermodynamics features in respect of association constant and standard free energy changes have been evaluated for both reactions. Both of methods have been proved to be very useful from routine quality control prospective and can be considered as superior to most of the published methods with respect to pace, ease, low cost, and sensitivity.

## 2. Experimental

### 2.1. Instrument

A UV-Visible Shimadzu Spectrophotometer 1601 with 1 cm path length quartz cells controlled by Shimadzu UV Probe 3.9 version software was used. MS Excel sheet was used to evaluate *F*, *t* tests, and other statistical parameters.

### 2.2. Materials and Reagents

Analytical grade reagents were used. Pregabalin and tranexamic acid pure drugs were a kind gift from a local pharmaceutical agency having 99% plus purity. Tranex capsules 250 and 500 mg and Syngab capsules 200 mg, 50 mg, and 100 mg (Atco Laboratories Ltd., Karachi, Pakistan) were procured from the market. TNP and DNP were acquired from Merck Sigma Aldrich, Germany. Microcrystalline cellulose (Avicel pH 101, maize starch, magnesium stearate, PVP, Aerosil R-200, crospovidone-X, HPMC (606), and PEG (6000)), titanium dioxide, and isopropyl alcohol were a gift from a local pharmaceutical agency. The entire chemicals were used according to their safety precautionary measures.

### 2.3. General Procedure

#### 2.3.1. Analytical Method Development

*Reagents and Standard Stock Solutions Preparation*. A 2.27 gL^−1^ TNP and 1.84 gL^−1^ 2,4-DNP solutions were prepared individually in dichloromethane corresponding to 10 mMol of the concerned reagent.

160 *μ*gmL^−1^ of PG and 155 *μ*gmL^−1^ of TXA were prepared individually by taking 16 and 15.5 mg weights, respectively, in 100 mL calibrated volumetric flasks, dissolved in 10 mL of water, and diluted to the mark level with the same solvent.

*Stoichiometric Study*. To study stoichiometry of the reaction, Job's method of continuous variation was followed [31]. Equimolar solution (1 mMol) of each of the coloring reagents and drugs were prepared. In a series of 10 mL calibrated amber volumetric flasks, solutions of PG and TXA were prepared individually, comprising different complementary proportions (0 : 10, 1 : 9, 2 : 8, 3 : 7, 4 : 6, 5 : 5,…, 9 : 1) each with both of the reagents (TNP and DNP) separately. After 10 minutes of reaction the absorbance of the complex was measured at the wavelength of maximum absorption against the reagent blanks treated similarly.

*DNP Method*. 3 mL of drug PG or TXA stock solution was taken individually in 10 mL amber volumetric flasks to which 4 mL of the coloring reagent DNP was added. Sample was diluted with acetonitrile up to volume. Absorbance was measured after 10 minutes of reaction at 418 nm using appropriate blank treated similarly.

*TNP Method*. 3 mL of drug PG or TXA stock solution was taken individually in 10 mL amber volumetric flasks to which 4 mL of the coloring reagent TNP was added. Acetonitrile was added up to the mark level. Absorbance was measured after 10 minutes of reaction at 425 nm using appropriate blank treated similarly.

#### 2.3.2. Procedures for Pharmaceuticals Formulation

Homogenous samples were obtained for each drug molecule. Bulk homogenous powder equivalent to 10 mg of pregabalin or tranexamic acid was dissolved in methanol by sonication for 15 min and shaking thoroughly for about 30–40 min. The samples were cooled down and diluted up to the mark level with methanol, mixed well, and filtered using a Whatman number 42 filter paper to give 100 *μ*gmL^−1^ of PG and TXA each, individually. Further dilution and reactions were continued as described above under general procedures.

#### 2.3.3. Excipients Interference Evaluation

10 mg of each PG and 10 mg of TXA were spiked individually with common excipients like magnesium stearate, HPMC (hydroxypropyl methylcellulose), glucose, pyrrolidone, lactose, talc powder, and starch. It was further processed as described under general procedures. Samples were subjected to multiple analyses and percent recovery was calculated.

#### 2.3.4. Reaction Mechanism

**Figure 2:**
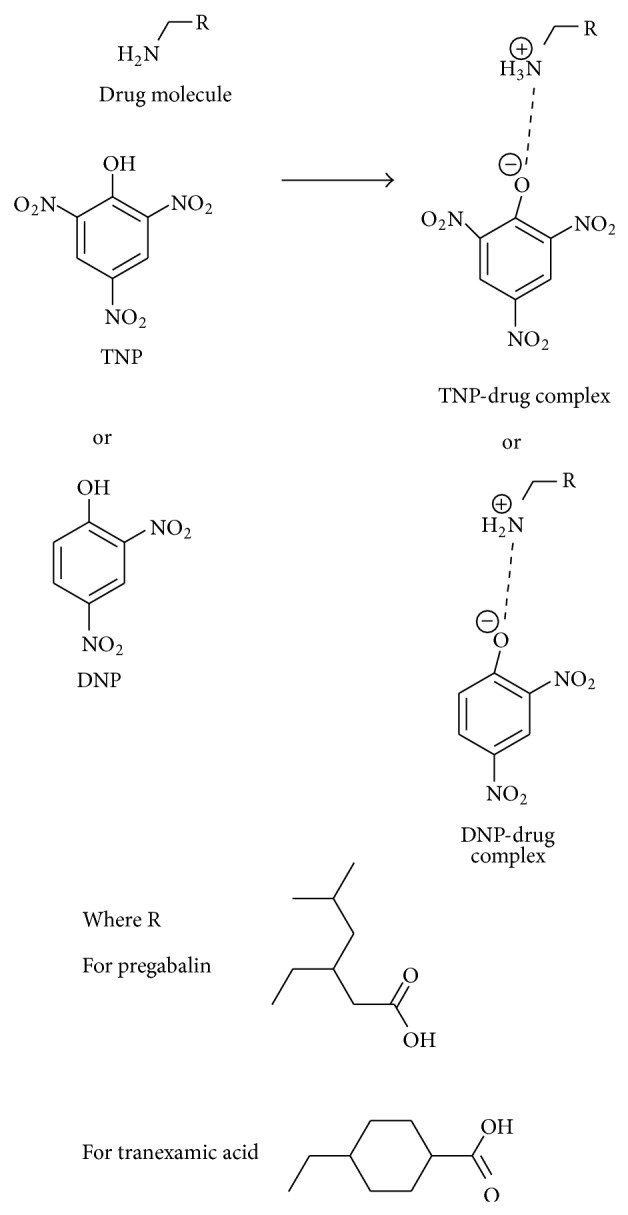
Mechanism of reaction.

This reaction is based on the proton transfer reaction from Lewis acid such as TNP and DNP to Lewis base such as TXA and PG, which produces intensely yellow color ion-pair complex as depicted by Saito and Matsunaga [26]. Mechanistic view has been expressed in Figure 2.

#### 2.3.5. Thermodynamic Studies

Standard free energy changes (Δ*G*°) and association constant (*K*_*c*_) were determined for both of the methods to evaluate thermodynamic aspects. The reaction of TNP and DNP each with PG and TXA was investigated for the association constants by the application of Benesi-Hildebrand equation [32]:

(1) $$\begin{array}{c} \frac{C_{a}}{A}=\frac{1}{\varepsilon }+\frac{1}{K_{c}\cdot \varepsilon }\cdot \frac{1}{C_{b}}, \end{array}$$

where *C*_*a*_ and *C*_*b*_ are the concentrations of the drug (PG or TXA) and coloring reagent (DNP or TNP) respectively, *A* is the absorbance of the complex, *ε* is the molar absorptivity of the complex, and *K*_*c*_ is the association constant of the complex.

Utilizing the above equation, *A* was plotted against *C*_*a*_ in both cases and straight lines were obtained. Standard free energy changes were calculated by the following equation:

(2) $$\begin{array}{c} {\Delta G}^{{}^{\circ}}=-2.356RT\log K_{c},\; \end{array}$$

where Δ*G*° is free energy change associated with complex (kJ mol^−1^), *R* is the thermodynamic gas constant (1.987 cal mol^−1^ deg^−1^), *T* is the Kelvin temperature (273+°C), and *K*_*c*_ is the equilibrium constant.

### 2.4. Assay Validation

ICH guideline was followed for the validation purpose and various experiments were performed [33].

#### 2.4.1. Linearity

Linearity is the ability of method to show absorbance correspondingly to the analytes concentration. Least square procedure was adopted to develop the regression equations which showed linear relation of the concentration of complex with absorbance, complying Lambert Beer's law. Under these experimental set-ups, absorbance at the given wavelength was found to vary directly with the concentrations of both the donner and the acceptor molecules.

#### 2.4.2. Precision and Accuracy

Accuracy of the analytical method is that the parameter confirms that the test results obtained with the method are close to the true or accepted values, while precision is the reproducibility of test result when the same homogenous sample is subjected to multiple testing. Solutions containing three different concentrations of pregabalin and tranexamic acid were arranged and investigated in triplicate for accuracy and precision.

#### 2.4.3. Specificity

Specificity is the quantification of the analytes in presence of component mixtures, excipients, and additives. The effect of common excipients and additives was evaluated by designing spiking experiments. Both TXA and PG were evaluated with common excipients at different concentration levels individually.

#### 2.4.4. Limit of Detection (LOD) and Limit of Quantification (LOQ)

Both reactions were evaluated for LOD and LOQ values. The empirical formulas 3*σ*/*S* and 10*σ*/*S* were used to establish LOD and LOQ for the method, where *S* is the slope and *σ* is the standard deviation of the response statistically inferred from calibration curve. A signal to noise ratio of 3 : 1 was defined for LOD and of 10 : 1 was defined for LOQ, respectively [33].

## 3. Results and Discussion

The reaction of coloring reagent TNP or DNP as Lewis acids with amino acid (PG and TXA) as Lewis base afforded intense yellow charge transfer complex. The intensity in color is attributed to the formation of phenolate ion [26] as shown in reaction scheme in Figure 2.

### 3.1. Method Optimization

**Figure 3:**
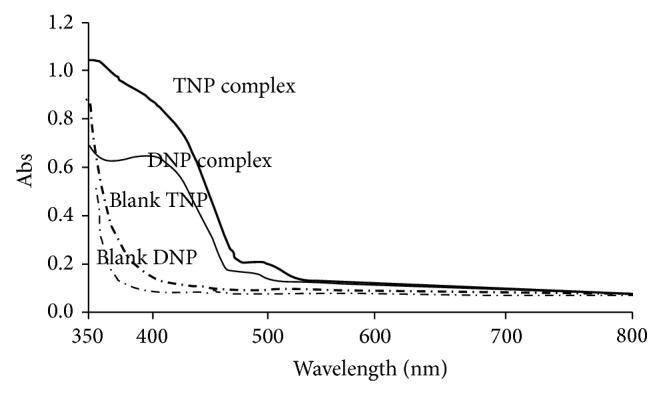
Spectrum of complexes and blanks.

In order to achieve optimum experimental condition, various factors were evaluated including time, temperature, solvent of choice, and concentration of drugs and reagent. Those experimental factors which were affecting the absorbing abilities of the resulting complex were optimized to enhance selectivity and sensitivity. The reaction and stability of the complex were evaluated with respect to time in intervals at room temperature. Maximum absorbance was obtained in 5- to 10-minute time period and prolonged period of time did not affect the absorbance of the complex. However, a negative impact was noted when reaction mixture was heated especially beyond 60°C. Similarly, effect of coloring reagent was evaluated by adding it to the fixed concentration of drug substance. To 100 *μ*gmL^−1^ of drug substance, reagents TNP and DNP were added in the range from 1 to 9 mL. The amount of coloring reagent linearly increased the absorbance up to equimolar stage beyond which the absorbance remains constant at maximum absorbance, which corresponds to 4.0 mL of solution of each DNP and TNP. Different solvents systems including chloroform, acetone, 2-propanol, acetonitrile, and dichloromethane were evaluated for optimum results. Water was used to prepare stock solution of PG and TXA because of the free solubility of the drugs and working range solutions were prepared in acetonitrile. It has been known that methanol interferes in charge transfer complex formation so acetonitrile was used in working range solutions in subsequent analysis [25]. Dichloromethane was found to be suitable for preparation of DNP and TNP solutions. The solutions were scanned in visible range (800 to 350 nm) by spectrophotometer using the corresponding blank. It was found that, for TNP complex, *λ*_max_ was 425 nm, while for DNP it was 418 nm which justifies intensity in color of complex derived from TNP as compared to that of DNP as in Figure 3. In view of these results, all working solutions to reaction were prepared in acetonitrile in sequence of drug-reagent solvent, maintained at 25 ± 2°C. Absorbance is measured at 418 and 425 nm for DNP and TNP complexes, respectively, after 10 minutes of mixing.

### 3.2. Stoichiometry

Job's continuous variations method suggested a 1 : 1 molar ratio for both reagents with both drugs. However, experimental design considered the drug molecules to be limiting reactants, so the chromogenic reagent was taken in slight excess in subsequent analysis in order to make the reaction drug concentration dependent and to counter any possible interference.

### 3.3. Thermodynamic Study of the Complex

Complexes were studied in detail by applying Bensi-hilbrand theory. Association constant (*K*_*c*_) was in the range of 1.51–1.62 × 10^3^ for DNP, while it was in the range of 2.74–2.86 × 10^3^ for TNP and standard free energy changes were −2.336 to −2.358 for complex derived from DNP and −2.498 to −2.508 for TNP-based complex. Results are portrayed in Table 1. Standard free energy changes value indicates spontaneity of the reaction. High wavelength, high association constant, and low standard free energy changes with complex involving TNP as compared to DNP justify the resonance phenomenon in the phenolate ion derived from TNP compared to DNP [26].

### 3.4. Validation Studies

After development method validation studies were conducted in line with the above-mentioned protocol. Using linear regression equation, methods showed linear response in concentration range from 0.02 to 200  *μ*g mL^−1^ with correlation coefficient of more than 0.9990 in both cases. In all the cases studied, Beer's law was obeyed with small intercept values (−0.0028 to 0.0021). Slopes were found to be in the range of 0.01018 to 0.0146. Small intercept and insignificant variation of slope are attributes of the excellent response of the methods. Low LOQ values of (0.095–0.109) *μ*g mL^−1^ explained sensitivity of the method as in Table 2. Obtained results in Tables 3 and 4 are very close to 100% label claim, indicating excellent accuracy, while very good precision is obvious from the % relative standard deviation which is less than 2. During spiking experiments no interference was found which indicated that none of those additives possess enough basicity to cause interference in analysis of TXA and PG. In addition, good % recoveries in common excipients spiked samples that ranged from 98.3 to 101.4% further confirmed the specificity of the projected methods.

### 3.5. Application of the Method in Pharmaceuticals

General procedures described above were followed to determine content of capsule dosage pharmulations for both of the drugs. The projected colorimetric methods based on charge-transfer complexes were applied to determine PG and TXA, along with the reference methods 9 and 15 for PG and TXA, respectively, to evaluate the test results. *t*-test and *F* test were carried out with MS Excel, and the proposed methods were found to be comparable to the referenced methods and no considerable variation was found between them which indicated similar precision and accuracy. Results are presented in Table 5.

## 4. Conclusions

To estimate the quantity of tranexamic acid and pregabalin in commercial products, two simple and sensitive colorimetric methods were developed and validated. Picric acid and 2,4-dinitrophenol, the two coloring reagents, have been utilized to develop two simple, much more common but sensitive and selective visible range spectrophotometric methods for routine analysis of tranexamic and pregabalin in bulk raw material and finished or semifinished dosage form. The suggested methods are superior to the already established spectrophotometric methods in terms of simplicity. *t* and *F* tests guaranteed high accuracy and precision. Hence, the proposed methods can be eagerly adopted by pharmaceutical quality control laboratories for routine quantitative analysis.

## Figures and Tables

**Table 1:** Thermodynamic study.

Drug molecule	DNP		TNP	
	PG	TXA	PG	TXA
Molar absorptivity (Ma)	1326.85	1337.35	1404.77	1418.36
*K*_*c*_	1.51 × 10^3^	1.62 × 10^3^	2.74 × 10^3^	2.86 × 10^3^
Δ*G*°	−2.336	−2.358	−2.498	−2.508

**Table 2:** Linearity and range.

Parameter	DNP		TNP	
	PG	TXA	PG	TXA
Linearity range (*μ*g/mL)	0.02–200	0.02–200	0.02–200	0.02–200
Correlation coefficient (*r*)	0.99962	0.99971	0.99948	0.99987
Slope (*m*)	0.01002	0.02035	0.01423	0.01553
Intercept (*c*)	−0.0012	−0.0028	0.0021	−0.0011
(LOD) (*μ*g/mL)	0.0059	0.0094	0.0041	0.0075
(LOQ) (*μ*g/mL)	0.0195	0.0302	0.0137	0.0269

**Table 3:** Accuracy and precision.

	Pregabalin (PG)			Tranexamic acid (TXA)		
	50% nominal	100% nominal	150% nominal	50% nominal	100% nominal	150% nominal
	con. %recovery	con. %recovery	con. %recovery	con. %recovery	con. %recovery	con. %recovery
	%l.c found %RSD			%l.c found %RSD		
DNP	98.10 1.235	101.68 0.525	101.93 0.235	99.84 0.545	99.84 0.412	101.85 0.864
TNP	100.60 0.320	99.89 0.536	99.17 0.938	101.60 0.940	100.69 0.526	101.57 0.638

**Table 4:** Recovery studies in commercial products.

Brand name (active molecule): Label claim	Label claim (%) ± S.D.	
	DNP	TNP
Tranex (TXA): 250 mg	101.23% ± 0.51	100.15% ± 0.61
Tranex (TXA): 500 mg	99.95% ± 0.63	99.19% ± 0.92

Syngab (PG): 100 mg	99.99% ± 0.77	99.91% ± 0.65
Syngab (PG): 200 mg	101.47% ± 1.13	100.27% ± 1.22

**Table 5:** *F* and *t* tests of the method.

Taken (mg)	TNP		DNP		Reference method	
	PG found (mg)	TXA found (mg)	PG found (mg)	TXA found (mg)	PG [9] found (mg)	TXA [15] found (mg)
100.1	100.2	100.6	101.6	100.7	100.3	101.1
101.1	99.8	100.3	98.6	99.4	100.8	99.8
100.2	100.1	99.8	100.9	100.3	99.7	101.2
100.4	100.2	99.6	99.1	98.7	99.3	100.7
100.3	100.6	100.6	98.4	97.9	99.5	100.4
99.6	100.5	101.4	97.5	98.1	100.5	100.3
MEAN	100.23	100.38	99.35	99.13	100.01	100.58
SD	0.288	0.646	1.576	1.184	0.601	0.527
RSD	0.287	0.644	1.586	1.195	0.601	0.524
*t*-Test	0.44	0.569	0.355	0.022		
*F*-Test	0.131	0.665	0.054	0.110		
